# Evaluation of Higher-Level Instrumental Activities of Daily Living via Micro-Doppler Radar Sensing of Sit-to-Stand-to-Sit Movement

**DOI:** 10.1109/JTEHM.2020.2964209

**Published:** 2019-01-07

**Authors:** Kenshi Saho, Kazuki Uemura, Masahiro Fujimoto, Michito Matsumoto

**Affiliations:** 1 Faculty of EngineeringToyama Prefectural University 57948 Imizu939-0398Japan; 2 Human Augmentation Research CenterNational Institute of Advanced Industrial Science and Technology 13508 Kashiwa277-0882Japan; 3 Department of Social WelfareToyama College of Welfare Science 157392 Imizu939-0341Japan

**Keywords:** Activity recognition, Doppler radar, motion analysis, statistical analysis

## Abstract

This paper presents an evaluation technique for higher-level instrumental activities of daily living (HL-IADLs), which are defined as relatively complicated modern daily activities to perform independently, using micro-Doppler radar (MDR) signatures of sit-to-stand-to-sit (STSTS) movements. Because HL-IADLs are useful for evaluating the degree of disability and cognitive decline in daily life, this study aims to develop a system that enables the identification of individuals with HL-IADL impairments in an unconstrained manner. The study participants were elderly adults of age 65–74 years of rural communities in Japan, and their motion parameters in natural STSTS were extracted via a single 24-GHz MDR installed on the ceiling. Their HL-IADLs were evaluated using a questionnaire-based scale called the Japan Science and Technology Agency Index of Competence (JST-IC). The relationship between the HL-IADLs scaled with the JST-IC and the extracted STSTS parameters were statistically analyzed, and the results revealed that the extracted parameters were associated with the JST-IC score. Furthermore, an appropriately accurate screening method was verified for elderly adults with HL-IADL impairment using the extracted parameters.

## Graphical abstract

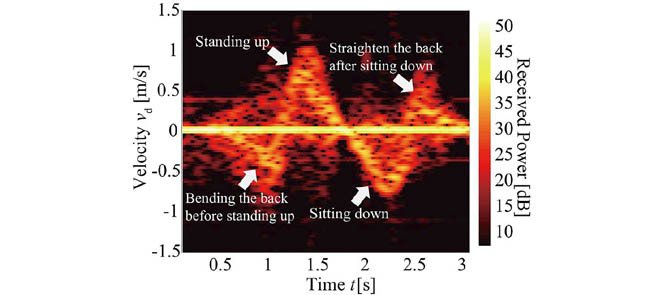

## I. Introduction

Evaluation of instrumental activities of daily living (IADLs), which are defined as relatively complicated daily tasks to perform independently (e.g., cooking, shopping, and using the phone), is important for the present aging society because the IADL score is sensitive to cognitive decline and physical disabilities [1]. Individual evaluation of IADLs would be useful to design effective treatment and rehabilitation plans. In recent years, the importance of higher-level IADLs (HL-IADLs) in modern society (e.g., mobile phone use, e-mails, and participating in neighborhood association events) has been noted because HL-IADLs require even higher-level competence than IADLs and the evaluation of such activities would be more effective in identifying frailty or risk of requiring care [2]. As reported in previous studies, it is possible to detect subtle cognitive decline by assessing HL-IADLs [3], [4]. Therefore, daily evaluation of HL-IADLs, to detect impairment in certain activities, is important to identify individuals with mild cognitive impairment.

To evaluate IADLs and HL-IADLs, questionnaire- based methods have been used. The most used evaluation scale is the Lawton IADL scale proposed in 1969 [5], and this is still the general method used today [6], [7]. IADL scales unique to each country have also been developed; examples include the Seoul IADL in Korea [8], an IADL scale for dementia intervention for elderly sub-Saharan Africans [9], and the Tokyo Metropolitan Institute of Gerontology Index of Competence (TMIG-IC) in Japan [10]. Although these scales have been widely used and validated, they are inspired by Lawton’s scale. Thus, these conventional scales do not include evaluation for HL-IADLs. To address this issue, scales to assess HL-IADLs have been studied recently [2]–[4]. One example of such a scale is the Japan Science and Technology Agency Index of Competence (JST-IC), developed in 2015 [2], [11], [12]. The JST-IC can effectively evaluate abilities to perform higher-level activities compared to the conventional IADL scales [2]. However, these IADL and HL-IADL scales are all questionnaire-based and thus not suitable for daily assessment of individuals.

For automatic IADL evaluation using daily monitoring, sensing-based approaches have recently been studied. The representative approach for this is smart home-based sensing [13]–[15]. In this approach, wireless pressure and infrared sensors are embedded in IADL-related equipment (phone, television, etc.) and places (kitchen, bathroom, etc.), which enables direct detection of IADLs. However, this approach is costly, cumbersome, and requires long-term monitoring to obtain rich information corresponding to the quality required for IADL scales. In addition, its accuracy is dependent on the number of sensors and their immediate environment. Relatively simple systems using wearable accelerometers have been proposed [16], but these require the subjects to wear devices for extended durations in a day. It is also difficult to directly measure activities related to HL-IADLs using these conventional techniques.

As a solution to the aforementioned problems, an indirect measurement of the IADL scale, based on the association between IADLs and daily motion parameters, can be considered. In physiotherapy and epidemiology, relationships between the IADL scales and some daily motion parameters are known, which allows us to investigate a sensing-based approach for IADL evaluation. For example, sit-to-stand-to-sit (STSTS) movement is considered to be an easily measurable motion in daily life, and correlations for STSTS movements with IADL scales have been reported [17]–[23]. For example, significant relationships have been reported between IADL (or more fundamental activities of daily living) dependence and strength in knee extension [17] and muscle mass [18], [19] related to STSTS movement. Thus, we can hypothesize that the magnitude of STSTS kinematic parameters (velocity, acceleration, etc.) of people with greater ability to conduct IADLs are relatively large. However, most studies have used a five-times sit-to-stand (5STS) test to assess the physical functions in STSTS movements [20], [21]; this test is unsuitable for daily monitoring because of the constrained environment and motions of the participants. This test requires five quick repetitions of the STSTS movements, and the movements must be measured by a professional such as a physiotherapist. Sensing-based techniques to assess STSTS movements, such as techniques involving the use of force plates [22] or accelerometers [23], [24], have been studied, but these techniques are unsuitable for daily use because of their limited measurement and installation capabilities.

To achieve automated daily measurements of STSTS, micro-Doppler radar (MDR) is a promising technique because it can remotely measure human motion without large and/or costly instruments and constrained subject environments [25], [26]. With its applicability to low-light conditions and to persons wearing ordinary clothes as its advantages, MDR has been investigated for use in home and hospital health monitoring applications in recent years. For example, our previous study [27] verified the effectiveness of an MDR-based gait analysis to assess cognitive functions. Li *et al.* [28] detailed a passive MDR system for e-Health applications including evaluations of physical activity and respiration, while Seifert *et al.* [29], [30] developed an MDR system for rehabilitation applications. Although some studies have done MDR measurements of STSTS, their objectives were to classify motion types including STSTS, walking, falling, and downward bending [26], [28], [31], [32]. Thus, measurement of STSTS motion parameters using MDR has not been undertaken. Moreover, to the best of our knowledge, there are no reports on radar-based IADL and HL-IADL evaluations.

This study presents an MDR-based measurement method of STSTS movement and investigates the associations of the measured STSTS parameters with IADL and HL-IADL scales for elderly adults. One of the novelties of this study is that it presents the first radar-based evaluation of IDALs. In addition, the technique is novel in terms of practical use as it provides unconstrained screening of individuals that require care for performing HL-IADLs. We first detail the MDR sensing and signal processing technique to extract STSTS motion parameters from elderly adults. The extracted motion parameters are times, velocities, accelerations, and jerks (time-derivative of acceleration) during one cycle of natural STSTS movement. Subsequently, relationships between the extracted parameters and the conventional IADL and HL-IADL scales are statistically evaluated. We show that there are significant associations between the extracted STSTS parameters and the HL-IADL scale, whereas no such significant associations are noted between the extracted STSTS parameters and conventional IADL scales. These findings also have novel implications for epidemiological studies on IADL evaluations. Finally, screening capability is demonstrated for participants with HL-IADL impairment, using the extracted parameters.

## II. Experimental Procedure

The study participants were 96 community-dwelling older adults aged 65–74 years (31 men and 65 women, mean age 69.9 ± 2.61 years, mean height 158.1 ± 8.65 cm, and mean mass 54.9 ± 9.31 kg). All participants were Japanese and could read and write the Japanese language. For all participants, the scores of a mini-mental state examination [33] were greater than 23 points, indicating that no participant has dementia. We also conducted other cognitive tests of the participants to investigate information processing speed, verbal fluency, and short-term memory functions using the same tests in [27], and we confirmed that all participants were cognitively healthy. All participants could perform the 5STS test without assistance.

All measurements were taken in a community setting. All participants performed the TMIG- and JST-IC. These were used as the ground truth of the conventional IADL and HL-IADL scales. Then, the participants performed the conventional 5STS and MDR-based STSTS tests. The results of these IADL and STSTS tests were statistically analyzed to develop the MDR-based IADL evaluation techniques. The concrete procedure of the experiments is as follows.

1) TMIG-IC test to measure conventional IADL scale [10]: The TMIG-IC is an effective conventional IADL scale developed in 1991 and primarily used in Japan; the English version is shown in Table 1 [10]. This test is a paper-based questionnaire with “Yes” or “No” answers. This study used the Japanese version of the TMIG-IC because all participants were Japanese. The TMIG-IC score is the number “Yes” answers, with the maximum score is 13 points.
2) JST-IC test to measure HL-IADL scale [11], [12]: The JST-IC is one of the new IADL scales and the English version of the JST-IC is shown in Table 2 [12]. Same as the TMIG-IC test, this is also a paper-based questionnaire with “Yes” or “No” answers. As with the TMIG-IC, we used its Japanese version. The score of the JST-IC is also the number of “Yes” answers, with a maximum score of 16 points. As indicated in Tables 1 and 2, the JST-IC is composed of more complex activities compared to the TMIG-IC (and other various conventional IADL scales such as Lawton’s scale [2]). In addition, it includes important activities of today’s society such as the usage of electronic devices and participation in the local community.
3) 5STS test [21]: The time to complete the 5STS test $T_{\mathrm {5sts}}$ is generally used in epidemiologic and physiotherapy studies; it is obtained through the results of a conventional STSTS measurement technique. This study used a straight back chair with a seat height of 0.43 m for the 5STS test. The participants were first asked to sit on the chair with their arms folded across their chests. They were then instructed to perform five repetitions of the standing-up and sitting-down motions as quickly as possible, while keeping their arms folded. The time from the start of motion to the fifth standing-up motion was measured as the result.
4) MDR-based STSTS measurement: The detailed kinematic parameters of the STSTS movement were obtained using our MDR system. The following section presents the system details.
5) The results of the MDR-based STSTS test, 5STS test, TMIG-IC, and JST-IC were statistically analyzed to investigate the effectiveness of using MDR to evaluate conventional and HL-IADL scales.

**TABLE 1:** TMIG-IC Test Sheet [10]

Questionnaires	Yes or No
Q1	Can you use public transportation (bus or train) by yourself?
Q2	Are you able to shop for daily necessities?
Q3	Are you able to prepare meals by yourself?
Q4	Are you able to pay bills?
Q5	Can you handle your own banking?
Q6	Are you able to fill out forms for your pension?
Q7	Do you read newspapers?
Q8	Do you read books or magazines?
Q9	Are you interested in news stories or programs dealing with health?
Q10	Do you visit the homes of friends?
Q11	Are you sometimes called on for advice?
Q12	Are you able to visit sick friends?
Q13	Do you sometimes initiate conversations with young people?

**TABLE 2:** JST-IC Test Sheet [12]

Questionnaires	Yes or No
Q1	Can you use a mobile phone?
Q2	Can you use the ATM?
Q3	Can you operate a video recorder such as a Blu-ray recorder or DVD player?
Q4	Can you send an e-mail using a mobile phone or computer?
Q5	Are you interested in news and events from overseas?
Q6	Can you determine the credibility of health-related information?
Q7	Do you enjoy art, films, or music?
Q8	Do you watch educational/cultural programs?
Q9	Do you follow any measures to prevent yourself from becoming a victim of crimes?
Q10	Do you try to be creative while doing daily tasks (cleaning, cooking)?
Q11	Can you take care of an ill person?
Q12	Do you take care of your grandchildren, family members, or acquaintances?
Q13	Do you participate in regional festivals or events?
Q14	Do you participate in a neighborhood association or a residents’ association?
Q15	Would you be able to assume a managerial position such as an organizer in a residents’ association or group activities?
Q16	Do you engage in charity or volunteer activities?

The experimental protocol was approved by the local ethics committee (Toyama Prefectural University, approval no. H29-1). Participants were provided with written and verbal instructions for test procedures, and written consent was obtained from each participant prior to testing.

## III. Micro-Doppler Radar (MDR) Measurement Technique for STSTS Movement

### A. Measurement Setup

**FIGURE 1.**
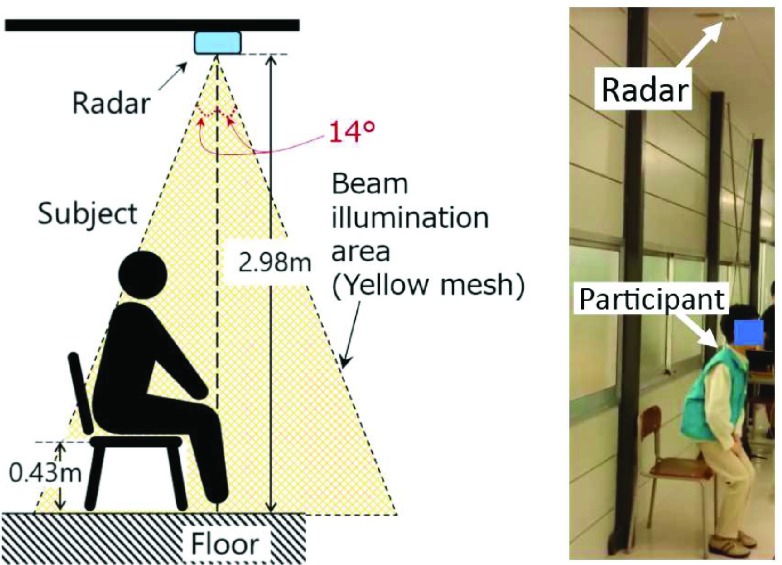
MDR measurement setup (left) and experimental site (right).

Figure 1 shows an MDR system for STSTS measurements and an experimental site. A single MDR was installed on a flat ceiling with a height of 2.98 m. The MDR position was just above the participant’s head when he/she was standing. The MDR transmitted a sinusoidal wave with a frequency of 24.0 GHz and an effective isotropic radiated power of 40 mW. The directivity of the MDR was ±14° and the radar beam was illuminated only near the chair as indicated in Fig. 1. This study assumed that only one participant is in the beam illumination area during the MDR measurement. A signal was obtained through demodulation of the reflected wave. The received signal is composed of the Doppler frequencies corresponding to the velocities of the scatters on each body part such as the head, arms, and legs. The sampling frequency of the received signal was 600 Hz, corresponding to a measurement velocity range of ± 1.875 m/s.

Aside from the 5STS test, we measured natural STSTS with the MDR to obtain effective results for use in daily monitoring. First, the participants were asked to sit in a chair with a seat height of 0.43 m and self-selected foot positions. They were then instructed to stand up and sit down at a self-selected speed. Only one cycle of these motions was measured. No restrictions were imposed on arm motion or types of clothes and shoes (none of the participants were wearing shoes with high heels).

### B. Time-Velocity Distribution (Spectrogram)

The STSTS motion parameters were extracted from the time-velocity distribution of the received signals. Therefore, similar to the procedure of [27], the variation in body part velocities over time was determined by obtaining the short-time Fourier transform (STFT) of the received signals. The received signal is defined as $s(t)$ and its STFT spectrogram $\vert S(t$, $f_{\mathrm {d}})\vert ^{2}$ was calculated, where $t$ is time and $f_{\mathrm {d}}$ is the Doppler frequency. The Hamming window function, which has a length of 128 samples (213 ms) was empirically used for the STFT process. The velocity $v_{\mathrm {d}}$ was calculated by taking the Doppler frequency $f_{\mathrm {d}}$ as $v_{\mathrm {d}} =$ *cf*_d_/($2f_{0}$), where $c$ is the speed of light and $f_{0} =24.0$ GHz is the frequency of the transmitting signal. Using this, we obtained the time-velocity distribution of the received signal $\vert S(t$, $v_{\mathrm {d}})\vert ^{2}$ corresponding to the STSTS of the participant.

**FIGURE 2.**
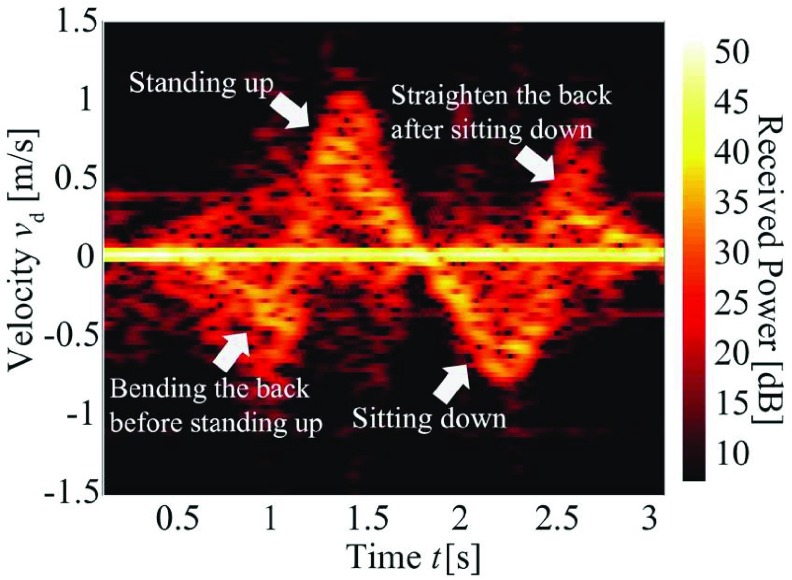
Representative spectrogram for the MDR received signal of STSTS.

Figure 2 shows an example of the time-velocity distribution (STFT spectrogram) of one cycle of the STSTS. The strongest echoes in $v_{\mathrm {d}} =0$ corresponded to the reflection from static targets such as the chair, wall, and floor. Characteristic components indicating the temporal variation of $v_{\mathrm {d}}$ with relatively strong received powers were confirmed. These components corresponded to the standing up and sitting down motions and related before or after motions (Fig. 2). Although significant peaks corresponding to body parts such as legs and arms [26], [27] were presented in the spectrogram, the components largely corresponded to echoes from the head because the radar was placed at the ceiling and the head was thus the closest body part.

### C. Method for Motion Parameter Extraction

The procedure to extract the STSTS motion parameters is as follows. Table 3 summarizes the sixteen extracted motion parameters and their definitions.

**FIGURE 3.**
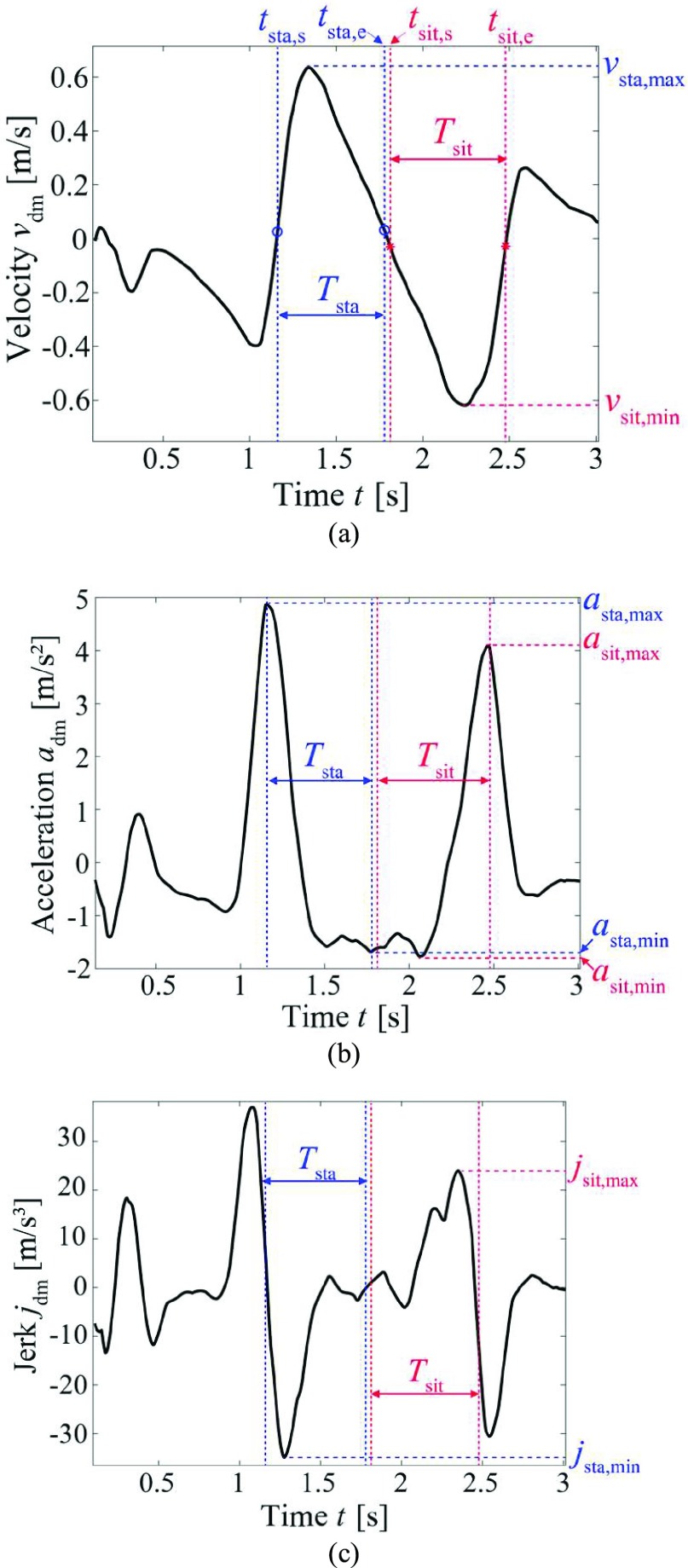
Extracted median Doppler frequency components from Fig. 2 and its time-derivatives. (a) Velocity $v_{\mathrm {dm}}(t)$ as extracted median Doppler frequency of the spectrogram, (b) acceleration $a_{\mathrm {dm}}(t)$ obtained by time-derivative of $v_{\mathrm {dm}}(t)$, (c) jerk $j_{\mathrm {dm}}(t)$ obtained by time-derivative of $a_{\mathrm {dm}}(t)$.

1) Detection of the STSTS movements is performed based on motion classification techniques [26], [28], [31], [32]. After the detection of the STSTS of individuals, the following processes are conducted to assess the kinematic parameters. In our experiments, this process was not conducted because the participants performed only STSTS movements (for practical use, this detection process is required).
2) High-pass filtering of the spectrogram was performed to remove the strong echoes from the static targets. We empirically used a one-dimensional Butterworth high-pass filter with a cut-off value of $v_{\mathrm {d}} =0.2$ m/s for each time.
3) The median Doppler frequency at each time $t$ was calculated to extract the time-series of the main components in the spectrogram. The median frequency defined in [34] was used. The extracted time variation of $v_{\mathrm {d}}$ is defined as $v_{\mathrm {dm}}(t)$. Figure 3(a) shows $v_{\mathrm {dm}}(t)$ extracted from the spectrogram of Fig. 2.
4) Maximum velocity in sit-to-stand and minimum velocity in stand-to-sit motions were extracted by $v_{\mathrm {sta, max}} \,\,=$ Max $v_{\mathrm {dm}}(t)$ and $v_{\mathrm {sit, min}} =$ Min $v_{\mathrm {dm}}(t)$. We also estimated $t_{\mathrm {vmax}}$ which is time corresponding to $v_{\mathrm {sta, max}}$.
5) Start and end times of sit-to-stand and stand-to-sit movements were determined. We defined the nearest time that satisfies $v_{\mathrm {dm}}(t) = \alpha v_{\mathrm {sta, max}}$ and $t < t_{\mathrm {vmax}}$ as the start time $t_{\mathrm {sta, s}}$ of the sit-to-stand motion, where dimensionless constant $\alpha =0.05$ was empirically selected. Similarly, the nearest time that satisfies $v_{\mathrm {dm}}(t) = \alpha v_{\mathrm {sta, max}}$ and $t > t_{\mathrm {vmax}}$ was the end time $t_{\mathrm {sta, e}}$ of the sit-to-stand motion. For the stand-to-sit movement, its start and end times were similarly calculated, and defined as $t_{\mathrm {sta, s}}$ and $t_{\mathrm {sta, e}}$. Using these time parameters, the durations of the sit-to-stand and stand-to-sit were calculated as $T_{\mathrm {sta}} = t_{\mathrm {sta, e}}$ - $t_{\mathrm {sta, s}}$ and $T_{\mathrm {sit}} = t_{\mathrm {sit, e}}$ - $t_{\mathrm {sit, s}}$, respectively (See Fig. 3(a)).
6) Mean velocities in the sit-to-stand and stand-to-sit motions were calculated as $v_{\mathrm {sta, mean}} =$ E[$v_{\mathrm {dm}}(t)$] (E[] indicates the mean with respect to $t$) for $t_{\mathrm {sta, s}} < t < t_{\mathrm {sta, e}}$ and $v_{\mathrm {sit, mean}} =$ E[$v_{\mathrm {dm}}(t)$] for $t_{\mathrm {sit, s}} < t < t_{\mathrm {sit, e}}$.
7) The time-derivative of $v_{\mathrm {dm}}(t)$ was calculated and defined as $a_{\mathrm {dm}}(t)$ (Fig. 3(b)). Using this, the acceleration parameters were extracted using a similar process for $v_{\mathrm {dm}}(t)$. For $t_{\mathrm {sta, s}} < t < t_{\mathrm {sta, e}}$, maximum, minimum, and mean accelerations in the sit-to-stand were calculated as $a_{\mathrm {sta, max}} =$ Max $a_{\mathrm {dm}}(t) a_{\mathrm {sta, min}} =$ Min $a_{\mathrm {dm}}(t)$, and $a_{\mathrm {sta, mean}} =$ E[$\vert $ $a_{\mathrm {dm}}(t)\vert $]. For $t_{\mathrm {sit, s}} < t < t_{\mathrm {sit, e}}$, $a_{\mathrm {sit, max}}$, and $a_{\mathrm {sit, mean}}$ were also calculated.
8) The time-derivative of $a_{\mathrm {dm}}(t)$ was calculated to determine jerk signal $j_{\mathrm {dm}}(t)$ (See Fig. 3(c)). The motion parameters related to the jerk were similarly extracted: $j_{\mathrm {sta, mean}}$ and $j_{\mathrm {sta, min}}$ were calculated for $t_{\mathrm {sta, s}} < t < t_{\mathrm {sta, e}}$ and $j_{\mathrm {sit, mean}}$ and $j_{\mathrm {sit, max}}$ were calculated for $t_{\mathrm {sit, s}} < t < t_{\mathrm {sit, e}}$.
9) The absolute values of all parameters were calculated and used for the statistical analysis.

**TABLE 3:** List of Extracted Motion Parameters in STSTS

Parameter	Definition
$T_{\mathrm {sta}}$	Duration for sit-to-stand
$v_{\mathrm {sta, mean}}$	Mean velocity in sit-to-stand
$v_{\mathrm {sta, max}}$	Maximum velocity in sit-to-stand
$a_{\mathrm {sta, mean}}$	Mean acceleration in sit-to-stand
$a_{\mathrm {sta, max}}$	Maximum acceleration in sit-to-stand
$a_{\mathrm {sta, min}}$	Minimum acceleration in sit-to-stand
$j_{\mathrm {sta, mean}}$	Mean jerk in sit-to-stand
$j_{\mathrm {sta, min}}$	Minimum jerk in sit-to-stand
$T_{\mathrm {sit}}$	Duration for stand-to-sit
$v_{\mathrm {sit, mean}}$	Mean velocity in stand-to-sit
$v_{\mathrm {sit, min}}$	Minimum velocity in stand-to-sit
$a_{\mathrm {sit, mean}}$	Mean acceleration in stand-to-sit
$a_{\mathrm {sit, max}}$	Maximum acceleration in stand-to-sit
$a_{\mathrm {sit, min}}$	Minimum acceleration in stand-to-sit
$j_{\mathrm {sit, mean}}$	Mean jerk in stand-to-sit
$j_{\mathrm {sit, max}}$	Maximum jerk in stand-to-sit

## IV. Evaluation With Statistical Analysis

### A. Analysis Methods

Associations between the motion parameters in Table 3 and the two IADL scales were statistically analyzed. We also examined the associations of the conventional 5STS results of $T_{\mathrm {5sts}}$ and compared them to the results of the proposed STSTS parameters. We first calculated Spearman’s correlation coefficients [35] of the motion parameters as well as the TMIG-IC and JST-IC scores, to clarify the STSTS parameters associated with the IADL and HL-IADL scales.

Subsequently, we investigated the statistical differences between the high and low score groups of the TMIG-IC and JST-IC. However, values to distinguish high/low scores have not been established for both IADL scales. Thus, we set the cutoff values based on the mean and standard deviation of the participant scores; we set the cutoff score as the mean minus standard deviation. $p$-values of Welch’s $t$-test were calculated and an effect size, Hedge’s g, was calculated to evaluate the magnitude of the differences. We set the significance level at $p =0.05$ and judged that $\vert $ *g* $\vert >0.5$ indicates a sufficiently large difference between the two groups based on the results of [36].

We also examined the effectiveness of the extracted motion parameters when screening HL-IADL impairment. Receiver operating characteristic (ROC) curves [34] that help investigate the screening accuracy for the JST-IC low-score group participants were depicted for the logistic regression model, which was achieved with the lowest Akaike information criterion (AIC) [38]. The lowest AIC method selects the appropriate combination of the STSTS parameters for the screening based on the minimization of information lost on the classification of two groups. We define the objective variable of the logistic regression $p_{\mathrm {imp}}$, which indicates the probability that the participant is in the low HL-IADL score group. The logistic model is expressed as

\begin{equation*}{\mathrm {log}}_{e}\left ({\frac {p_{imp}}{1-p_{imp}} }\right) =\beta _{0} + \beta _{1}x_{1}+ \beta _{2}x_{2} + \beta _{3}x_{3}+\cdots,\tag{1}\end{equation*}

where $x_{i}$ is $i$-th selected parameter with the lowest AIC method and $\beta _{i}$ is $i$-th coefficient of the model. We depict a ROC curve using $p_{\mathrm {imp}}$. The area under the curve (AUC) for this model was compared with that for the model used only for $T_{\mathrm {5sts}}$, to verify the effectiveness of the derived motion parameters. The significance of the difference between AUCs was evaluated using $p$-values obtained from DeLong’s test [37], with a significance level of $p =0.05$.

### B. Results and Discussion

#### 1) Correlation Analysis

**FIGURE 4.**
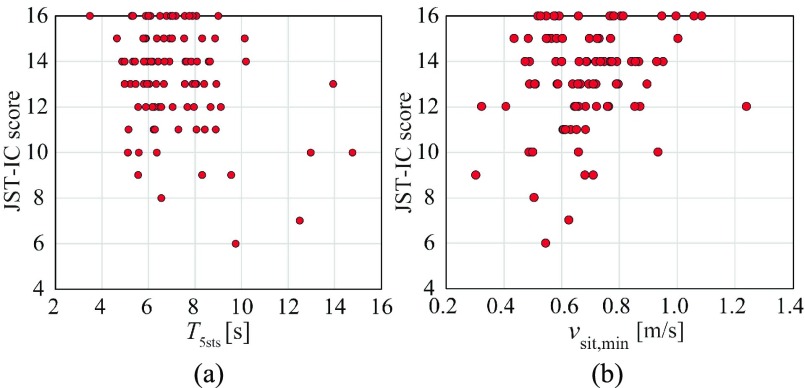
Relationship between JST-IC score and: (a) $T_{\mathrm {5sts}}$, (b) $v_{\mathrm {sit, min}}$.

Table 4 lists the Spearman’s correlation coefficients of the STSTS parameters, as well as the TMIG-IC and JST-IC scores along with their $p$-values. No correlations to the TMIG-IC were found in all STSTS motion parameters including $T_{\mathrm {5sts}}$. In contrast, $v_{\mathrm {sta, max}}$ and $v_{\mathrm {sit, min}}$ were weakly correlated to the JST-IC ($\rho \ge0.2$ with $p < 0.05$). Very weak correlations to the JST-IC were also found for a few parameters ($\rho >0.15$ with $p < 0.1$). No correlations were found between the $T_{\mathrm {5sts}}$ and the JST-IC. Figure 4 shows the relationship between $T_{\mathrm {5sts}}$, $v_{\mathrm {sit, min}}$, and the JST-IC score of all participants. Similar correlations to those summarized in Table 4 are observed for $v_{\mathrm {sit, min}}$ in Fig. 4.

**TABLE 4:** Spearman’s Correlation Coefficients of SiStSi Motion Parameters to IADL Scales and Their P-Values

	TMIG-IC		JST-IC	
	$\rho $	${p}$-value	$\rho $	${p}$-value
$T_{\mathrm {5sts}}$ [s]	−0.138	0.182	−0.156 ^*^	0.133
$T_{\mathrm {sta}}$[s]	−0.0459	0.657	−0.0638	0.539
$v_{\mathrm {sta, mean}}$ [m/s]	−0.136	0.187	0.148	0.154
$v_{\mathrm {sta, max}}$[m/s]	−0.100	0.332	0.217 ^**^	0.0350 ^‡^
$a_{\mathrm {sta, mean}}$[m/s^2^]	−0.0633	0.540	0.148	0.153
$a_{\mathrm {sta, max}}$[m/s^2^]	−0.0624	0.546	0.200 ^**^	0.0595 ^†^
$a_{\mathrm {sta, min}}$[m/s^2^]	0.0439	0.671	0.152 ^*^	0.141
$j_{\mathrm {sta, mean}}$[m/s^3^]	−0.0139	0.893	0.160 ^*^	0.122
$j_{\mathrm {sta, min}}$[m/s^3^]	0.0285	0.783	0.177 ^*^	0.0869 ^†^
$T_{\mathrm {sit}}$ [s]	−0.0160	0.988	−0.083	0.424
$v_{\mathrm {sit, mean}}$[m/s]	−0.102	0.324	0.149	0.149
$v_{\mathrm {sit, min}}$[m/s]	−0.0325	0.753	0.222 ^**^	0.0305 ^‡^
$a_{\mathrm {sit, mean}}$[m/s^2^]	−0.0254	0.806	0.172 ^*^	0.0959 ^†^
$a_{\mathrm {sit, max}}$[m/s^2^]	−0.0115	0.912	0.0509	0.624
$a_{\mathrm {sit, min}}$[m/s^2^]	−0.127	0.219	0.194 ^*^	0.0595 ^†^
$j_{\mathrm {sit, mean}}$[m/s^3^]	0.0138	0.894	0.162 ^*^	0.117
$j_{\mathrm {sit, max}}$[m/s^3^]	0.0130	0.899	0.174^*^	0.0927 ^†^

^*^ : very weak correlation of $0.15 < \rho < 0.2$ and

^**^ : weak correlation of $\rho \ge0.2$.

^†^ : $p < 0.1$ and

^‡^ : $p < 0.05$.

These results indicated that the most of STSTS parameters obtained by our MDR system were correlated with HL-IADL scale of the JST-IC, but not with the conventional IADL scale of the TMIG-IC. For associations between the conventional IADL scales and the 5STS test, a few studies have reported only weak correlations [22], while others report no correlations [39], [40]; our results for the TMIG-IC are consistent with these conventional studies. In contrast, we newly confirmed that the JST-IC (HL-IADL scale) was significantly associated with the STSTS motion parameters measured by the MDR.

#### 2) Statistical Differences in High/Low Score Groups

This subsection discusses the results of Welch’s $t$-test to investigate the significance of differences between high and low score groups of TMIG-IC and JST-IC. First, we set cutoff values to form these groups. The TMIG-IC and JST-IC scores of all subjects were 12.4 ± 1.15 and 13.3 ± 2.24 points, respectively. For both scales, the mean minus standard deviation was approximately 11 points. Therefore, we set a cutoff value of 11/12 for both TMIG-IC and JST-IC to distinguish low/high score groups.

**FIGURE 5.**
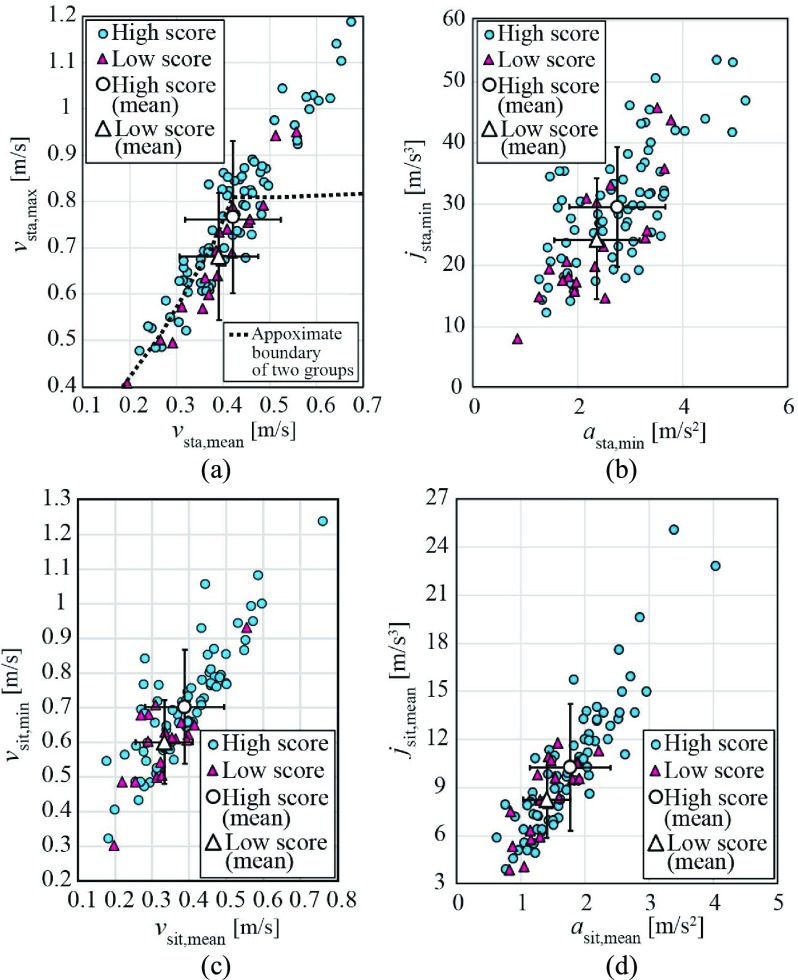
Examples of the extracted parameters for all participants: (a) ($v_{\mathrm {sta, mean}}$, $v_{\mathrm {sta, max}}$), (b) ($a_{\mathrm {sta, min}}$, $j_{\mathrm {sta, min}}$), (c) ($v_{\mathrm {sit, mean}}$, $v_{\mathrm {sit, min}}$), (d) ($a_{\mathrm {sit, mean}}$, $j_{\mathrm {sit, mean}}$).

Table 5 summarizes the results of the statistical difference evaluation for the TMIG-IC and indicates that there were no significant differences between the two groups for all STSTS parameters. Table 6 summarizes the results for the JST-IC. The absolute values of $v_{\mathrm {sta, max}}$, $j_{\mathrm {sta, min}}$, $v_{\mathrm {sit, mean}}$, $v_{\mathrm {sit, min}}$, $a_{\mathrm {sit, mean}}$, $a_{\mathrm {sit, min}}$, and $j_{\mathrm {sit, mean}}$ of the high-score groups were significantly larger than those of the low-score groups, with a sufficient effect size of $\vert $ *g* $\vert >0.5$. The overlaps of 95 % confidence interval (95 % CI) of the two groups were small for these parameters. Fig. 5 shows plots of the STSTS parameters; Fig. 5(a) includes an approximate boundary to screen the low-score group. These figures also indicate significant differences between the two groups even though a definitive boundary was not confirmed. As shown in Fig. 5(a), although the differences in $v_{\mathrm {sta, mean}}$ between the two groups is not significant (Table 6), we can find a boundary for screening in this plane as indicated by the dashed line in this figure, through which the screening accuracy can be improved over using only $v_{\mathrm {sta, max}}$. This means that the combination of these parameters is effective for the screening of low score groups. A similar tendency is confirmed in other parameters in Fig. 5 through which screening of low-score participants could be achieved, as described in the next subsection. Note that there were no differences between men and women for all results.

**TABLE 5:** Results for Differences for High/Low Score Groups of TMIG-IC

	High score (≥12) Mean ± SD	Low score (<12) Mean ± SD	$p$ (*: <0.05)	$\vert \text{g}\vert $ (*: >0.5)
Information of participants				
Number	82 (23 men)	14 (8 men)		
Age (years)	70.0 ± 2.62	69.8 ± 2.63	0.843	0.06
Height (m)	157.5 ± 8.35	161.6 ± 9.83	0.160	0.47
Mass (kg)	54.0 ± 8.89	60.3 ± 10.2	0.0434*	0.69 *
5STS test				
$T_{\mathrm {5sts}}$(s)	7.01 ± 1.76	7.83 ± 2.53	0.260	0.43
MDR measurement				
$T_{\mathrm {sta}}$(s)	0.722 ± 0.165	0.734 ± 0.292	0.886	0.06
$v_{\mathrm {sta, mean}}$ (m/s)	0.407 ± 0.0908	0.464 ± 0.132	0.137	0.58*
$v_{\mathrm {sta, max}}$(m/s)	0.739 ± 0.151	0.811 ± 0.211	0.238	0.45
$a_{\mathrm {sta, mean}}$(m/s^2^)	2.05 ± 0.653	2.37 ± 0.943	0.235	0.46
$a_{\mathrm {sta, max}}$(m/s^2^)	4.18 ± 0.988	4.53 ± 1.35	0.368	0.33
$a_{\mathrm {sta, min}}$(m/s^2^)	2.63 ± 0.850	2.98 ± 1.19	0.305	0.39
$j_{\mathrm {sta, mean}}$(m/s^3^)	12.4 ± 4.71	13.5 ± 6.14	0.512	0.23
$j_{\mathrm {sta, min}}$[m/s^3^)	28.2 ± 9.51	29.3 ± 12.7	0.772	0.1
$T_{\mathrm {sit}}$ (s)	0.805 ± 0.171	0.786 ± 0.173	0.710	0.11
$v_{\mathrm {sit, mean}}$(m/s)	0.367 ± 0.0945	0.438 ± 0.136	0.082	0.69 *
$v_{\mathrm {sit, min}}$(m/s)	0.676 ± 0.150	0.719 ± 0.218	0.486	0.27
$a_{\mathrm {sit, mean}}$(m/s^2^)	1.67 ± 0.539	1.89 ± 0.900	0.371	0.38
$a_{\mathrm {sit, max}}$(m/s^2^)	3.30 ± 0.942	3.69 ± 1.57	0.386	0.36
$a_{\mathrm {sit, min}}$(m/s^2^)	2.40 ± 0.741	2.82 ± 1.16	0.214	0.51 *
$j_{\mathrm {sit, mean}}$(m/s^3^)	9.73 ± 3.38	10.7 ± 5.57	0.543	0.25
$j_{\mathrm {sit, max}}$(m/s^3^)	22.2 ± 7.56	22.2 ± 8.71	0.990	0

**TABLE 6:** Results for Differences for High/Low Score Groups of JST-IC

	High score (≥12)		Low score (<12)		$p$ (*: < 0.05)	$\vert \text{g}\vert $ (*: >0.5)
	Mean ± SD	95% CI	Mean ± SD	95% CI		
Information of participants						
Number	76 (25 men)		19 (5 men)			
Age (years)	69.9 ± 2.55	69.3–70.5	70.0 ± 2.87	68.6–71.4	0.842	0.05
Height (m)	159.0 ± 8.32	156.9–160.7	154.1 ± 8.92	149.8–158.4	0.0388 *	0.41
Mass (kg)	55.7 ± 9.10	53.6–57.7	51.8 ± 9.97	47.0–56.6	0.138	0.40
5STS test						
$T_{\mathrm {5sts}}$(s)	6.92 ± 1.52	6.58–7.27	8.08 ± 2.79	6.74–9.43	0.0953	0.63*
MDR measurement						
$T_{\mathrm {sta}}$(s)	0.721 ± 0.165	0.683–0.758	0.741 ± 0.264	0.614–0.868	0.759	0.1
$v_{\mathrm {sta, mean}}$ (m/s)	0.420 ± 0.102	0.396–0.443	0.391 ± 0.0865	0.349–0.433	0.206	0.3
$v_{\mathrm {sta, max}}$(m/s)	0.766 ± 0.164	0.727–0.801	0.681 ± 0.141	0.613–0.749	0.0300 *	0.53 *
$a_{\mathrm {sta, mean}}$(m/s^2^)	2.13 ± 0.719	1.97–2.29	1.92 ± 0.663	1.60–2.24	0.224	0.3
$a_{\mathrm {sta, max}}$(m/s^2^)	4.33 ± 1.05	4.09–4.56	3.85 ± 0.973	3.38–4.32	0.0666	0.46
$a_{\mathrm {sta, min}}$(m/s^2^)	2.75 ± 0.918	2.53–2.95	2.36 ± 0.839	1.96–2.77	0.0876	0.43
$j_{\mathrm {sta, mean}}$(m/s^3^)	12.8 ± 4.80	11.7–13.9	11.3 ± 5.47	8.72–14.0	0.285	0.3
$j_{\mathrm {sta, min}}$(m/s^3^)	29.5 ± 9.74	27.3–31.7	24.2 ± 10.1	19.3–29.0	0.0436 *	0.55 *
$T_{\mathrm {sit}}$ (s)	0.790 ± 0.169	0.751–0.827	0.851 ± 0.179	0.765–0.937	0.188	0.36
$v_{\mathrm {sit, mean}}$(m/s)	0.387 ± 0.107	0.362–0.411	0.332 ± 0.0796	0.294–0.371	0.0169 *	0.53 *
$v_{\mathrm {sit, min}}$(m/s)	0.702 ± 0.165	0.663–0.738	0.601 ± 0.124	0.541–0.661	0.00532 *	0.64 *
$a_{\mathrm {sit, mean}}$(m/s^2^)	1.77 ± 0.632	1.63–1.91	1.40 ± 0.381	1.22–1.59	0.00223 *	0.62 *
$a_{\mathrm {sit, max}}$(m/s^2^)	3.42 ± 1.10	3.17–3.67	3.05 ± 0.819	2.66–3.45	0.115	0.34
$a_{\mathrm {sit, min}}$(m/s^2^)	2.55 ± 0.867	2.35–2.74	2.11 ± 0.517	1.86–2.34	0.00667 *	0.54 *
$j_{\mathrm {sit, mean}}$(m/s^3^)	10.2 ± 3.95	9.36–11.1	8.25 ± 2.46	7.07–9.44	0.00825 *	0.54 *
$j_{\mathrm {sit, max}}$(m/s^3^)	22.9 ± 7.84	21.1–24.7	19.3 ± 6.76	16.1–22.6	0.0581	0.46

Similar to the results of the correlation analysis, these results also showed the significant associations of our STSTS motion parameters with the JST-IC and no significant associations of those with the TMIC-IC. Table 7 summarizes the findings in this paper. Although the conventional 5STS test results of $T_{\mathrm {5sts}}$ are not efficient to evaluate both the conventional and HL-IADLs, the kinematic parameters of the STSTS movement obtained using the MDR measurements are effective to identify the participants with HL-IADL impairment; the reason for and mechanism of these results are discussed in Section V-B.

**TABLE 7:** Summary of Significance in Relationship Between IADL Scales and STSTS Tests

	TMIG-IC (Conventional IADLs)	JST-IC (HL-IADLs)
5STS test (Conventional)	Not significant	Not significant^*^
MDR STSTS test (Proposed)	Not significant^*^	Significant^*^

^*^ denotes the novel results revealed in this paper

#### 3) Screening Capability for HL-IADL Impairment

**FIGURE 6.**
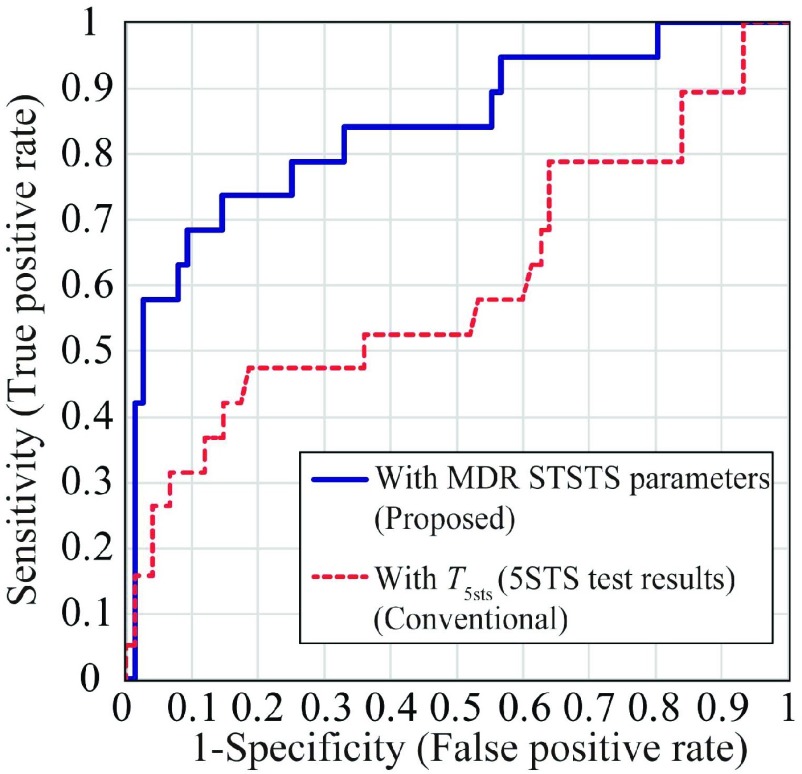
ROC curves for the classification of high/low score groups of JST-IC with the proposed and conventional techniques.

Figure 6 shows ROC curves for screening the low score-group of the JST-IC, using the conventional $T_{\mathrm {5sts}}$ and logistic regression results $p_{\mathrm {imp}}$ with our MDR STSTS parameters. The STSTS parameters ($v_{\mathrm {sta, mean}}$, $v_{\mathrm {sta, max}}$, $a_{\mathrm {sta, min}}$, $j_{\mathrm {sta, \textrm {}mean}}$, $j_{\mathrm {sta, min}}$, $j_{\mathrm {sit, mean}}$) were selected for the logistic regression of Eq. (1) with the lowest AIC method. The coefficients of these parameters were (30.3, −13.8, −2.25, 0.650, 0.221, −0.255) with the intercept of $\beta _{0}= 2.31$. $v_{\mathrm {sta, mean}}$ is selected for screening according to the discussion in the previous section because the lowest AIC method selects the combination of parameters that can be effectively used for the screening. The AUCs of the MDR STSTS parameters and $T_{\mathrm {5sts}}$ were 0.842 and 0.602, respectively. DeLong’s test resulted in $p \,\,=0.020$, indicating significant differences in the AUCs of the two methods. These results demonstrated that the MDR STSTS parameters achieved better screening accuracy than the conventional 5STS test result of $T_{\mathrm {5sts}}$. Thus, the kinematic parameters of the STSTS velocity, acceleration, and jerk were effective to screen HL-IADL impairment; the reasons for this are discussed in Section V-C from biomechanical aspects.

## V. Overall Discussion

### A. Contributions

The objective of this study was to develop an MDR-based evaluation technique for conventional and HL-IADLs. From the viewpoint of radar technology, this study is significant as the development of the first MDR-based HL-IADL sensing technique. Thus far, no remote and unconstrained sensing techniques for evaluating IADLs (and HL-IADLs) have been reported. Furthermore, the proposed technique uses only the STSTS movement and does not require large observation space. Thus, the proposed evaluation system is applicable for not only rural communities but also clinics and homes. Thus, this study promotes the development of health monitoring systems for elderly people under various situations with its unconstrained and narrow-space capabilities.

The other contribution is the validation of our hypothesis described in the Introduction for the HL-IADLs: the magnitude of the STSTS parameters of people with greater ability to conduct IADLs was large. The statistical analyses in the previous section verified this hypothesis for the HL-IALD scale of elderly people aged 65–74 years. However, we also verified that our STSTS kinematic parameters were ineffective for the evaluation of conventional IDALs. These results summarized in Table 7 provide important findings that would be effectively used for not only clinical and daily health monitoring applications, but also future epidemiological studies to investigate the relationships between health status and IADLs, HL-IADLs, and advanced concepts of IADLs that will be proposed for the future society.

### B. Reason for Differences in the Results for Conventional and HL-IADL Scales

We discuss the results summarized in Table 7; i.e., the reason for the extracted STSTS parameters being associated with the HL-IADLs scaled with the JST-IC, but not with the conventional IADLs scaled with the TMIG-IC. To this end, we focus on the difference in the questionnaires between the TMIG-IC (Table 1) and the JST-IC (Table 2).

It is evident that the JST-IC requires complex physical and intellectual activities in social groups and this should be related to the physical function required to establish STSTS movement. Although Q10–13 of the TMIG-IC cover abilities regarding simple visitation and conversations, Q13–16 of the JST-IC require activities that deeply relate to social groups (e.g., participation in festivals and resident associations). The former can be performed with a light physical load, but the latter requires relatively large physical activity. Significant relationships between social roles and physical functions have been reported in recent studies [41], [42]. Thus, unlike conventional IADLs, HL-IADLs scaled with the JST-IC appear to require higher-level physical functions that are reflected in the kinematic parameters of the STSTS.

The other HL-IADLs corresponding to the questions of the JST-IC require a relatively high level of physical and cognitive functions. Q9–12 of the JST-IC are related to life management and taking care of others. Sufficiently high cognitive and physical functions are required to safely perform these activities [43], [44]. In addition, these activities are complex compared with the simple IADLs in Q1–5 of the TMIG-IC. Although Q1–8 of the JST-IC do not require high-load physical activities, they require higher-level cognitive function [45], [46] (e.g., for mobile phone use) compared with Q6–9 of the TMIG-IC. It is accepted that the sit-to-stand motion is associated with both physical and cognitive function [47], [48]. Thus, the lack of physical and cognitive functions for performing HL-IADLs should be reflected in the kinematic parameters of STSTS movements.

### C. Biomechanical Aspects of the Results

Next, we discuss why the kinematic parameters of the STSTS are related to HL-IADL scale based on biomechanical aspects. To this end, the relationship between each kinematic parameter (velocity, acceleration, and jerk) and the results of associations to the HL-IADLs are considered. We first focus on the STSTS velocity. As discussed in the previous subsection, HL-IADL scaled with the JST-IC require high-level physical and cognitive functions. Many studies have reported that cognitive functions are significantly related to leg muscle mass and balance ability (fall risk) as well as physical functions [49], [50]. The relationship between balance ability and STSTS velocities have also been reported in biomechanics studies [51]. These studies have indicated the significant associations between physical/cognitive impairment (leading to lower HL-IADL ability) and less muscle mass and balance ability (leading to lower STSTS velocity). This can be attributed to the requirement of muscle strength for mobility, which is an important prerequisite for HL-IADLs (and conventional IADLs) [17]. Muscle mass is closely related to muscle strength and the ability to perform STSTS movements [18], [19]. With respect to acceleration, a few studies reported that accelerations in sit-to-stand motion are associated with the difficulty the elderly have in performing STSTS [23], [52] which could explain the relationship between STSTS acceleration and HL-IADL. Moreover, the minimum jerk has been reported to be an important factor for natural and smooth sit-to-stand motion [23], [53]. This may indicate that the participants with lower HL-IADL score have impaired control of limb movements to perform natural and smooth STSTS movement. Therefore, information on the high-dimensional physical/cognitive impairment was obtained as the variation in the kinematic parameters of the STSTS movement.

### D. Limitations

There are four limitations of this study. First, the participants’ age range was limited to 65–74 years. Because IADL and HL-IADL scales strongly depend on age, the results for participants aged 75 years and older might be different from our results. Future work should involve a comprehensive analysis that includes older adults aged 75 and older.

Second, all participants were Japanese, meaning that HL-IADL differences due to culture and race were not considered in our analysis. Although a few HL-IADL (also known as the advanced ADL) scales have been proposed [3], [4], simultaneous measurements with the STSTS, using the MDR, have not been conducted. Therefore, our results are still the first to exhibit potential for the diagnosis of HL-IADL impairment via STSTS movement measurements. Our results should be validated in other countries to help develop advanced monitoring systems for the elderly.

The third limitation is related to the simplicity of our MDR measurements and parameter extraction. In our MDR experiments, a single radar was installed on the ceiling. Using multiple radars, interallied in various places, can improve the accuracy of the HL-IADL evaluation. Furthermore, the extracted STSTS parameters were simple kinematic variables such as velocity and acceleration. High- dimensional parameters obtained using machine-learning approaches were not considered because the number of participants is insufficient for such techniques. Furthermore, high-resolution time-frequency analyses were not considered. These advanced parameter extraction and classification algorithms might be capable of diagnosing IADL impairments more accurately. However, our technique has important merits, such as the low-cost single MDR. Moreover, it does not disrupt the activities of daily life because of its small physical size, simple implementation, and non-contact measurements. Furthermore, our STSTS parameters represent the physical meanings of the head motion in the STSTS movement, and can be used to investigate the relationship between HL-IADL impairments and details of the physical disability status. Overall, while our study is limited by the simple MDR setting and parameter choice, the simplicity can be an advantage as well.

Finally, the fourth limitation is that we assumed a situation with only one participant in the beam illumination area of the MDR. For practical use in daily situations, the existence of more than one individual must be considered even when a relatively narrow beam illumination area can be used. Although a radar-based technique for multiple human separation [54] appears to be capable of solving this problem, it was not considered in this study, and its application is an important future task.

## VI. Conclusion

This study demonstrates the significant associations between the JST-IC of elderly participants in the age range 65–74 years and MDR-measured STSTS parameters toward unconstrained evaluation of HL-IADLs. Our results revealed that kinematic parameters of the STSTS are associated with HL-IADLs scaled with the JST-IC, but not with conventional IADLs scaled with the TMIG-IC. Furthermore, the conventional time to complete the 5STS test was not associated with either JST-IC or TMIG-IC. Thus, we demonstrated that STSTS kinematic parameters (velocity, acceleration, and jerk) measured with the MDR can effectively evaluate HL-IADLs. We verified the screening of the low-score group of the JST-IC with a sufficiently high AUC of 0.842. Thus, this study suggests that MDR is a promising candidate for the unconstrained detection of HL-IADL impairment; this can be effectively used for the early detection of individuals that require care to perform HL-IADLs.

Resolving the limitations described in the previous section should be the basis of our future work to develop practical monitoring systems for the elderly. In particular, application to state-of-the-art statistical analysis techniques, such as deep learning, based on data collected from larger numbers of participants is important. With such techniques, we can use not only echoes from the head but also motion information from other body parts such as arms and legs including as shown in Fig. 2. Thus, we can expect a significant improvement in the screening accuracy when employing the deep learning technique based on the rich information in the spectrogram. Moreover, in situations where other sensors such as cameras and accelerometers can be used, their sensor fusion with the MDR might improve the accuracy of the motion measurements and HL-IADL evaluations.
